# The effects of stress across the lifespan on the brain, cognition and mental health: A UK biobank study

**DOI:** 10.1016/j.ynstr.2022.100447

**Published:** 2022-04-14

**Authors:** Elizabeth McManus, Hamied Haroon, Niall W. Duncan, Rebecca Elliott, Nils Muhlert

**Affiliations:** a The University of Manchester, Division of Neuroscience & Experimental Psychology, UK; b Taipei Medical University, Graduate Institute of Mind Brain and Consciousness, Taiwan

**Keywords:** Stress, Cognition, Mental health, Brain microstructure, Brain volume

## Abstract

Repeated overstimulation of the stress response system, caused by exposure to prolonged highly stressful experiences, is thought to affect brain structure, cognitive ability, and mental health. We tested the effects of highly stressful experiences during childhood and adulthood using data from the UK Biobank, a large-scale national health and biomedical study with over 500,000 participants. To do this, we defined four groups with high or low levels of childhood and/or adulthood stress. We then used T1-and diffusion-weighted MRI data to assess the macrostructure of grey matter and microstructure of white matter within limbic brain regions, commonly associated with the stress response. We also compared executive function and working memory between these groups. Our findings suggest that in females, higher levels of Childhood stress were associated with reduced connectivity within the posterior thalamic radiation and cingulum of the hippocampus. In males however, higher levels of Adulthood stress is associated with similar changes in brain microstructure in the posterior thalamic radiation and cingulum of the hippocampus. High stress in Childhood and Adulthood was associated with decreases in executive function and working memory in both males and females. Stress across the lifespan was also positively associated with the number of diagnosed mental health problems, with a stronger effect in females than in males. Finally, our findings also suggest that cognitive and mental health outcomes due to stress may be mediated by the sex specific stress related changes in brain microstructure. Together our findings demonstrate clear links between stress at distinct phases of the lifespan, changes in measures of brain microstructure, impairments in cognitive abilities and negative mental health outcomes.

## 1 Introduction

Cumulative life stress is the accumulation of repeated exposure to stressful experiences across the lifespan. During childhood, highly stressful events (such as abuse or the loss of a parent) can impact an individual's cognitive abilities and both physical and mental health decades later as adults (Shonkoff et al., 2012; Hedges and Woon, 2011). Similarly, stressful events during adulthood, such as separation, bereavement or financial difficulties, are amongst the most influential risk factors for coronary heart disease (Yusuf et al., 2004), and are associated with greater cortisol reactivity to psychosocial stressors (Roos et al., 2018). Stressful life events also impact on cognitive abilities, such as memory and processing speed (Foong et al., 2018; VonDras et al., 2005). The timing at which stress occurs, either during periods of rapid brain development early in life or later, during periods of relative brain stability, may differentially impact upon brain structure to influence cognitive abilities and health. Here we assess the relative effects of childhood and adulthood stress on brain macro- and micro-structure, cognition and mental health diagnoses in a large cohort, using data from the UK Biobank (https://www.ukbiobank.ac.uk). Although explored as a whole cohort, we also explore these effects separately for males and females to identify any potential differences in outcomes given established variation in stress response between sexes (Kirschbaum et al., 1992; Bale and Epperson, 2015).

During childhood, overexposure to stress hormones can result in dysregulation of the immune system, leaving individuals vulnerable to infection and to disrupted development of the brain (Audage and Middlebrooks, 2008). For instance, sexual abuse experienced during childhood has been associated with reduced hippocampal volume (Andersen et al., 2008). Additionally, neglect and low socioeconomic status during childhood have been associated with reduced regional brain volumes (Frodl et al., 2010; Staff et al., 2012), such as the volume of grey matter (Teicher et al., 2012) and white matter (Frodl et al., 2010) in the hippocampus. Reductions in regional brain volumes associated with childhood stress are still detectable much later in life, suggesting a persistent influence of childhood stress on the brain (Frodl et al., 2010). Stress during adulthood is also associated with lower hippocampal volumes (Smith, 2005; Bremner, 1999; Woon et al., 2010; Schoenfeld and Gould, 2012) potentially due to disrupted patterns of neurogenesis (Mirescu and Gould, 2006). Critically, this is likely to differ by sex, with clear differences observed in both hippocampal function (Yagi and Galea, 2019) and in hormonal responses to stress (Oyola and Handa, 2017) in men and women. The comparative effects of childhood compared to adulthood stress both across the population and in men and women specifically, however, is not yet well understood.

In addition to brain volume alterations, changes in microstructure may occur in connected white matter tracts. Diffusion MRI can probe the barriers to water diffusion in the brain non-invasively and *in vivo*; it is sensitive to a loss of those barriers, such as through loss of myelin or axonal membranes. High levels of childhood stress are associated with reduced directionality of diffusion in white matter regions including the corpus callosum and uncinate fasciculus (Frodl et al., 2010; Paul et al., 2008; Jackowski et al., 2008; Eluvathingal et al., 2006). Reduced white matter integrity has been shown in adults who report high levels of life stress (Bergamino et al., 2016). In sum stress, either during child- or adulthood, can impact on the microstructure of the brain.

Cognitive abilities, such as memory, rely on regions of the limbic system such as the hippocampus and amygdala, which coincidentally have a high density of receptors for stress-related hormones (Roozendaal et al., 2009; Kim and Diamond, 2002; Vedhara et al., 2000; Johnson et al., 2007). As such, links between acute stress and worse cognitive performance (including divided attention and working memory) have been established across a wide range of studies (LeBlanc, 2009). Similarly, high levels of perceived stress predict the frequency of everyday cognitive failures (such as forgetting appointments etc), demonstrating the real-world impact of stress on cognition (Boals and Banks, 2012). This negative impact on cognition can either occur from residual effects of stress during childhood, affecting working memory and higher-order complex functions (Evans and Schamberg, 2009; Bremner and Narayan, 1998), or from stressors during adulthood (Aldwin and Levenson, 2001). Stress-related changes in these cognitive abilities may be underpinned by consequent alterations in limbic structures, such as the hippocampus and amygdala (Pechtel and Pizzagalli, 2011).

Mental health outcomes in later life are also linked to stressful experiences in childhood and adulthood (Paus et al., 2008; Lupien et al., 2009). A history of childhood trauma is associated with smaller hippocampal volumes, as seen in those with stress-related mental health problems including major depression and post-traumatic stress disorder (Neumeister et al., 2005; Villarreal et al., 2002; Vythilingam et al., 2002). In addition, major depression has been linked to reduced microstructural integrity in the posterior thalamic radiation (Liao et al., 2013). This damage or atrophy to microstructure in individuals with major depression has also been linked to stress (Lee et al., 2002). This suggests that stressful experiences across the lifespan likely impact on both cognition and mental health via their effects on brain structure.

Sex differences in stressor prevalence may also differentially impact upon consequent biological effects. Prevalence rates of extreme stressors, such as sexual and emotional abuse, are much higher in females than males (Walby and Towers, 2018; Dobash and Dobash, 2004). Stress hormones, such as cortisol, may also impact males and females differently due to differing levels of oestrogen and testosterone interacting with cortisol (Seeman et al., 2001; Kudielka et al., 2004). For example, animal models demonstrate that changes in gonadal hormones during ageing, particularly in females, interact with the regulation of genes relevant to stress reactivity (Bale and Epperson, 2015; McEwen, 2001; Shanmugan and Epperson, 2014; Suda et al., 2008). This increased stress responsivity is accompanied with alterations in limbic brain structures, providing a route through which sex differences in stress responsivity could affect not only brain structure, but also cognitive abilities and mental health outcomes commonly associated with these regions.

Using the full UK Biobank sample (*n* = 502,520), we first identified four specific categories of individuals based on high or low levels of stress experienced in childhood and/or adulthood. We examined the relative impacts of stress on brain structure, both grey matter regional volumes and white matter microstructure, in key regions of the limbic system. We predicted that those with high levels of childhood and adulthood stress would show reduced grey matter volume in these limbic regions. We additionally predicted altered white matter microstructure between these regions. Finally, we predicted that those with high childhood and adulthood stress would be more prone to worse cognitive performance and increased numbers of mental health diagnoses. Finally, we aimed to examine whether any group level effects are consistent across the full range of stress levels and between males and females when examined individually.

## 2 Methods

### 2.1 Participants

All participant datasets for this study were obtained from the UK Biobank from data released in February 2020 (https://www.ukbiobank.ac.uk/). The UK Biobank is a large-scale study collecting health and biomedical data from 500,000 generally healthy participants across Great Britain, aged 40–69 years between 2006 and 2010. A subset was later invited to a second visit to undergo brain MRI. Ethical approval was granted to the UK Biobank by the North West Multi-Centre Research Ethics Committee (REC reference 11/NW/0382) and all provided informed consent to participate and for their anonymised data to be used. The current study was conducted under approved UK Biobank application number 49224.

### 2.2 Defining stress groups

As part of the assessment at the UK Biobank, participants completed several online follow-up questionnaires. Before defining stress groups, participants with neurological conditions were removed from the dataset. We then estimated childhood stress or adulthood stress using the following questions: Childhood stress was assessed using five questions (Field ID 20489–20491) relating to how well-loved and looked after participants were as children, each answered on a five-point rating scale with reverse coding where necessary (see Appendix A). These questions were based on a shortened version of the Childhood Trauma Questionnaire (CTS-5) (Glaesmer et al., 2013), a modified version of an established and widely used measure of adverse life experiences occurring during childhood (Bernstein and Fink, 1998). These variables have also been used as a measure of childhood adverse life events in previous research using UK Biobank data (Gheorghe et al., 2021). Adulthood stress was measured using a similar set of five questions (Field ID 20521–20525) relating to the experience of potentially abusive relationships experienced as an adult, also using a five-point scale (see Appendix B). Scores of total childhood and adulthood stress were calculated as the sum of each of these five questions respectively. Participants with missing data on these questionnaires were excluded from the analysis (*n* = 147,131).

Once scores for childhood and adulthood stress had been calculated, groups for high and low childhood and adulthood stress were defined. Due to a floor effect leading to a severe skew in the number of participants scoring the lowest possible scores for both childhood and adulthood stress, criteria for low groups was set to scores of 0. As there are no set criteria for defining “high levels” of trauma/stress our *a priori* criteria for high stress was set as 2 standard deviations above the mean (childhood stress = 6.55, adulthood stress = 7.05). Using these criteria, we were able to define our four stress groups: 1) low childhood & adulthood stress, 2) low childhood but high adulthood stress, 3) high childhood but low adulthood stress and 4) high childhood and adulthood stress. Individuals scoring above 0 but lower than 2 standard deviations above the mean were not categorised into one of these groups and as such were not included in any group level analyses.

**Fig. 1:**
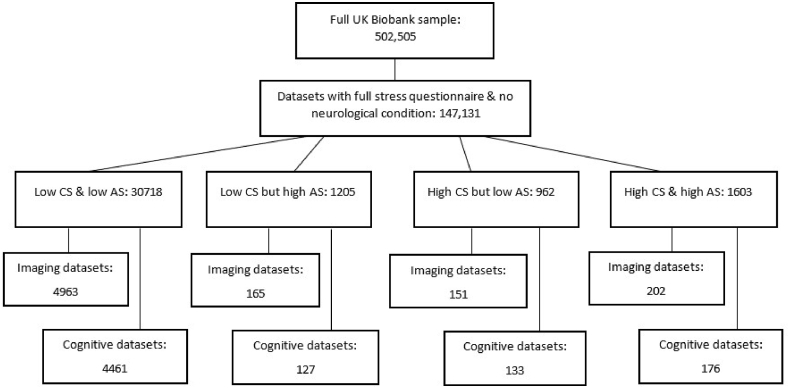
Flow chart demonstrating sample size at each stage of group selection.

Not all participants had all relevant data for both imaging and cognitive task analyses. Separate samples were therefore created for imaging and cognitive function analyses. Fig. 1 shows a flow chart of how group sizes were determined.

### 2.3 Brain image acquisition & processing

Full details of the MRI protocol and processing steps have previously been reported (Alfaro- Almagro et al., 2018; Miller et al., 2016). The UK Biobank used three dedicated imaging centres, each equipped with identical MR scanners (3.0 T Siemens Skyra, software VD13) using the standard Siemens 32-channel receive head coil. 3D MPRAGE T1-weighted volumes were both pre-processed and analysed by the UK Biobank imaging team using FSL (https://fsl.fmrib.ox.ac.uk/fsl/fslwiki) tools and analysis pipelines adapted from the Human Connectome Project (Miller et al., 2016; Jenkinson et al., 2012; Glasser et al., 2013). The current project takes advantage of the UK Biobank imaging team's release of analysed imaging data regional statistics, known as Imaging Derived Phenotypes (IDP) (Alfaro- Almagro et al., 2018).

#### 2.3.1 Regional volume analyses

**Fig. 2:**
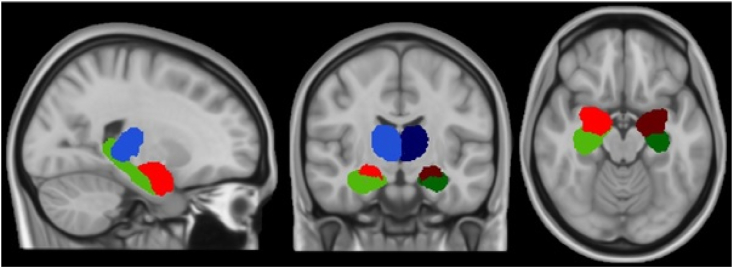
Regional volume maps for amygdala (red), hippocampus (green) and thalamus (blue) in the right (lighter shades) and left (darker shades) hemispheres. (For interpretation of the references to colour in this figure legend, the reader is referred to the Web version of this article.)

To compare regional volumes, this study used IDP summary values generated by the UK Biobank imaging team (Alfaro- Almagro et al., 2018). After pre-processing, these IDPs were created using FSL tools (FIRST) to generate regional grey matter volumes (Patenaude et al., 2011). The available limbic structures analysed in the current study were left and right hippocampus, amygdala and thalamus. These regions are shown in Fig. 2. All volumes were scaled for head size, estimated from intracranial volume (Voevodskaya et al., 2014).

#### 2.3.2 Microstructural analyses

**Fig. 3:**
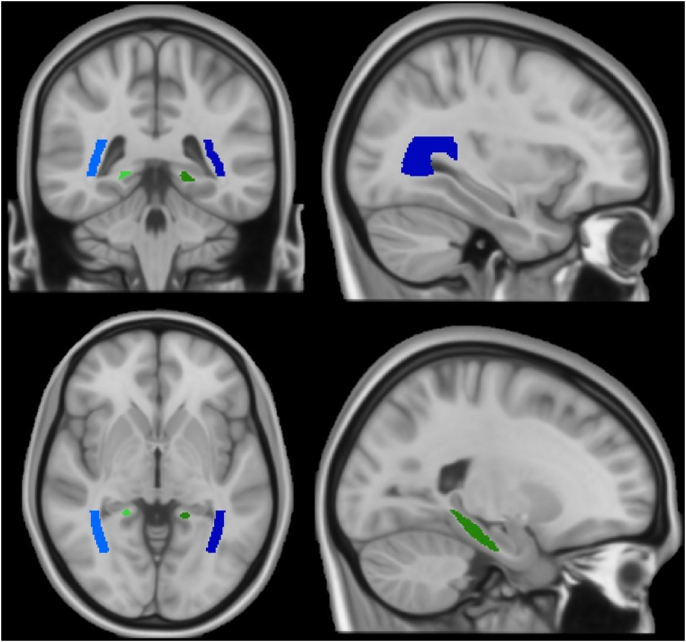
White matter region maps for cingulum of the hippocampus (green) and posterior thalamic radiation (blue) in the right (lighter shades) and left (darker shades) hemispheres. (For interpretation of the references to colour in this figure legend, the reader is referred to the Web version of this article.)

Diffusion MRI metrics were pre-processed and analysed by the UK Biobank imaging team (Alfaro- Almagro et al., 2018). These IDPs include DTI and NODDI measures in major white matter tracts. DTI fitting provides values for mean diffusivity (MD), fractional anisotropy (FA), diffusion tensor mode (MO) and eigenvalues (L1, L2 & L3), whereas the NODDI model provides neurite orientation dispersion (OD) in addition to intra-cellular volume fraction (ICVF, i.e. neurite density) and isotropic volume fraction (ISOVF). All diffusion metrics were analysed between groups in white matter regions: left and right posterior thalamic radiation and cingulum of the hippocampus (see Fig. 3). No diffusion data were available for the amygdala.

### 2.4 Cognitive function tasks

All cognitive function tasks were performed on a touchscreen during each participant's assessment centre visits. Scores for five of these tasks were analysed in this study: numeric memory, prospective memory, symbol digit substitution, trail-making and word production (see (Lyall et al., 2016) for procedures). These tasks demonstrate good concurrent validity and test-retest reliability (Fawns-Ritchie and Deary, 2020).

### 2.5 Mental health reporting

As part of the online follow-up questionnaires, participants were asked to report if they had ever had mental health problems diagnosed by a professional (Data Field 20544). Summative scores were calculated to determine how many diagnosed mental health problems each participant had experienced.

### 2.6 Statistical analysis

The handling of raw UK Biobank data, creation of sub samples and groups and the statistical analysis for all comparisons were all conducted using R (R Core Team, 2013). Individuals reporting neurological conditions or with missing data for measures of interest in relevant sub samples were excluded. Kruskal Wallis and chi-squared tests were used to compare groups’ ages and sex ratios, respectively.

To compare brain volumes, diffusion metrics and cognitive scores in the stress groups defined above, between-subjects ANOVA tests were used. Age and sex were added as covariates, resulting in the use of between-groups ANCOVA tests. We used false discovery rate (FDR) multiple comparisons corrections to adjust significance values (*p*-values), and only report significant values that reach this threshold. *Post hoc* pairwise comparisons were used to identify the direction of any significant group differences. To confirm these findings in the full UK Biobank sample (i.e. not just subgroups) we then used multiple regression analyses using all the appropriate data. We looked for relationships between stress and brain structure in male and female samples separately. Predictors used in these regressions were childhood stress, adulthood stress and age.

Due to missing data, cognitive comparisons were only possible for symbol digit substitution and trail making (both numeric and alphanumeric) tasks. We used the same ANCOVA models as the brain imaging data, with childhood and adulthood stress as predictors, adjusting for age and sex. *Post hoc* pairwise comparisons determined group differences. As with the brain structure analyses, we used multiple regression to explore the impact of stress on cognitive abilities in all available UK Biobank data for males and females separately. The predictors used within the regression analyses were childhood stress, adulthood stress and age. A final, further multiple regression analyses assessed the impact of stress on diagnosed mental health problems in males and females separately.

To explore if any changes in cognitive ability and mental health outcomes may be related to potential changes in brain structure, post hoc mediation analyses were performed in the males and females separately. These analyses tested whether the identified brain structure changes mediated the relationship between childhood or adulthood stress and either cognitive ability or mental health outcomes.

## 3 Results

### 3.1 Group differences: age & sex

Groups differed significantly in age (Kruskal Wallis: imaging [*χ*^*2*^(3) = 31.9, *p* < .001], cognitive data [*χ*^*2*^(3) = 30.96, *p* < .001]), and sex (Chi-square: imaging [*χ*^*2*^(3) = 134.2, *p* < .001], cognitive data [*χ*^*2*^(3) = 125.4, *p* < .001]; Table 1).

**Table 1:** Number of participants, mean ages (with standard deviation in parentheses) and sex ratios for each stress group across both imaging and cognitive datasets. Sex ratio is female to male.

	Brain imaging			Cognitive Data		
	*N*	Age	Sex	*N*	Age	Sex
Group 1 (Low Childhood & Low Adulthood stress)	4963	55.16 (7.16)	1.07	4461	55.16 (7.29)	1.04
Group 2 (Low Childhood & High Adulthood stress)	165	53.54 (7.30)	3.85	127	52.92 (7.44)	3.88
Group 3 (High Childhood & Low Adulthood stress)	151	54.07 (7.93)	1.7	133	53.37 (7.71)	1.89
Group 4 (High Childhood & High Adulthood stress)	202	52.66 (7.24)	5.5	176	52.91 (7.58)	5.77

### 3.2 Stress on brain volume

No significant volume differences were seen between groups for any of the six regions of interest (left and right hippocampus, amygdala and thalamus). As expected, both age and sex had large significant effects on both hippocampal and thalamic volume (for each, *p* < .0001), but not amygdala volume.

### 3.3 Stress on brain microstructure

Age was a significant covariate for all diffusion metrics in the posterior thalamic radiation. Sex was a significant covariate in bilateral FA, MD, L1 & ICVF, and right MO but not for ISOVF or OD in the posterior thalamic radiation.

**Fig. 4:**
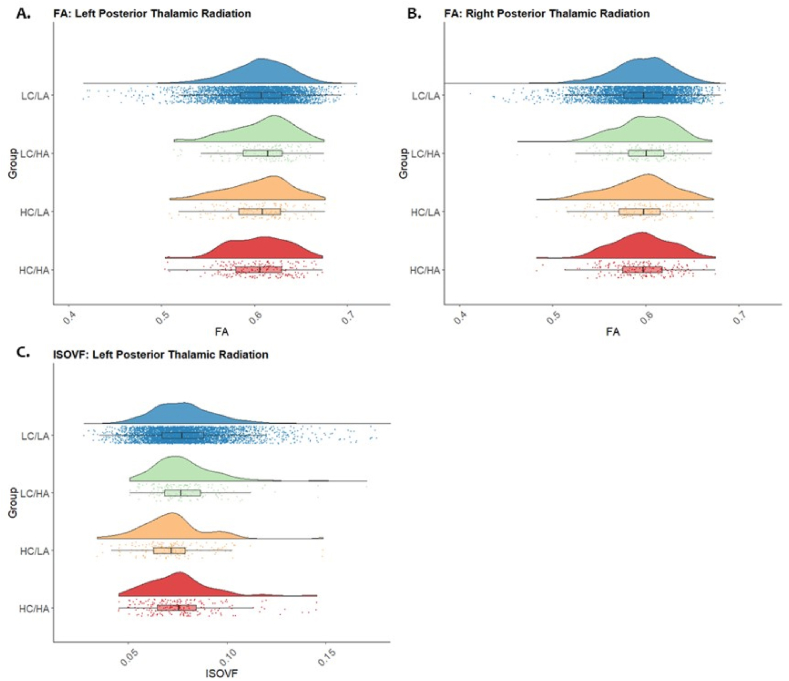
Raincloud plots representing data distributions in each of the four stress groups for values of FA in the left (A) and right (B) posterior thalamic radiation & ISOVF in the left (C) posterior thalamic radiation between each stress group. Box plots represent median and interquartile ranges of these values. Low Childhood and Low Adulthood = LC/LA, Low Childhood and high Adulthood = LC/HA, High Childhood and Low Adulthood = HC/LA and High Childhood and High Adulthood = HC/HA.

Significant group effects were seen in the right and left posterior thalamic radiation for FA (*F*(3, 5475) = 4.47, *p* = .014, *η*_*p*_^*2*^ = 0.002, & *F*(3,5475) = 4.02, *p* = .014, *η*_*p*_^*2*^ = 0.002) and left posterior thalamic radiation for ISOVF (*F*(3, 5475) = 5.42, *p* = .004, *η*_*p*_^*2*^ = 0.003). Data distributions for FA and ISOVF in bilateral posterior thalamic radiation can be seen in Fig. 4.

*Post hoc* pairwise comparisons revealed reduced FA in the right and left posterior thalamic radiation for High Childhood/High adulthood compared to the Low Childhood/Low Adulthood stress group (right: *p* = .018, 0.008% reduction in mean FA, Left: *p* = .009, 0.11% reduction in mean FA). The High Childhood/Low Adulthood group also showed significantly lower left posterior thalamic radiation ISOVF compared to Low Childhood/Low Adulthood (*p* = .0005, 7.88% reduction mean ISOVF), LC/HA (*p* = .003, 8.24% reduction in mean ISOVF), and High Childhood/High Adulthood stress (*p* = .035, 5.73% reduction in mean ISOVF) groups.

No significant group differences in diffusion were seen in the left or right cingulum of the hippocampus. Age predicted all diffusion metrics in the cingulum of the hippocampus, except for MO on the right side. Additionally, sex predicted FA, MO, ICVF, L2 and ISOVF of the left and right cingulum of the hippocampus, MD, L1 and L3 in the right, and OD in the left cingulum of the hippocampus.

### 3.4 Whole UK biobank brain data

Regression analyses were then used to explore if the group effects of stress on the brain are still seen in the wider UK Biobank sample. Given the significant effects of sex in the above analyses, regressions were run separately for the male and female samples. All male (N = 11,290) and female (N = 13,285) participants with appropriate stress and imaging data were included in these regressions. Age was a significant predictor for all diffusion measures in both the male and female samples. Additionally, age was a significant predictor of hippocampal and right amygdala volumes in both males and females. Volumes of the thalamus and left amygdala were not significantly predicted by age for either sex.

Adulthood and Childhood stress did not significantly predict regional brain volumes in either males or females. In the male sample, Adulthood, but not Childhood, stress significantly predicted changes in diffusion metrics in the left posterior thalamic radiation and bilateral cingulum of the hippocampus (see Table 2). In females, only Childhood stress significantly predicted diffusion measures in the left cingulum of hippocampus and bilaterally in the posterior thalamic radiation (see Table 3).

**Table 2:** Multiple regression outputs for significant diffusion metrics with childhood and adulthood and stress in the male sample only.

		Childhood stress		Adulthood stress		Overall model fit	
Imaging Metric	Region	β	P	β	p	R^2^	p
FA	Left posterior thalamic radiation	−0.011	.348	−0.028	.010*	0.108	<.001*
MO	Left posterior thalamic radiation	0.014	.317	−0.025	.034*	0.056	<.001*
OD	Left posterior thalamic radiation	−0.001	.879	0.030	.008*	0.030	<.001*
ISOVF	Left cingulum of the hippocampus	−0.016	.132	0.030	.004*	0.044	<.001*
	Right cingulum of the hippocampus	−0.013	.193	0.039	<.001*	0.016	<.001*

**Table 3:** Multiple regression outputs for significant diffusion measures in the brain with childhood and adulthood and stress in the female sample only.

		Childhood stress		Adulthood stress		Overall model fit	
Imaging Metric	Region	β	P	β	p	R^2^	p
FA	Left posterior thalamic radiation	−0.037	<.001*	−0.019	.140	0.0951	<.001*
	Right posterior thalamic radiation	−0.039	<.001*	−0.014	.203	0.0990	<.001*
MD	Left cingulum of the hippocampus	0.027	.006*	−0.001	.961	0.0112	<.001*
	Left posterior thalamic radiation	0.023	.014*	0.005	.961	0.0876	<.001*
	Right posterior thalamic radiation	0.031	.002*	0.000	.961	0.0804	<.001*
ICVF	Left cingulum of the hippocampus	−0.025	.009*	−0.009	.961	0.0312	<.001*
	Left posterior thalamic radiation	−0.040	<.001*	−0.009	.551	0.0667	<.001*
	Right posterior thalamic radiation	−0.042	<.001*	−0.004	.336	0.0533	<.001*

### 3.5 Impact of childhood and adulthood stress on cognitive function

**Fig. 5:**
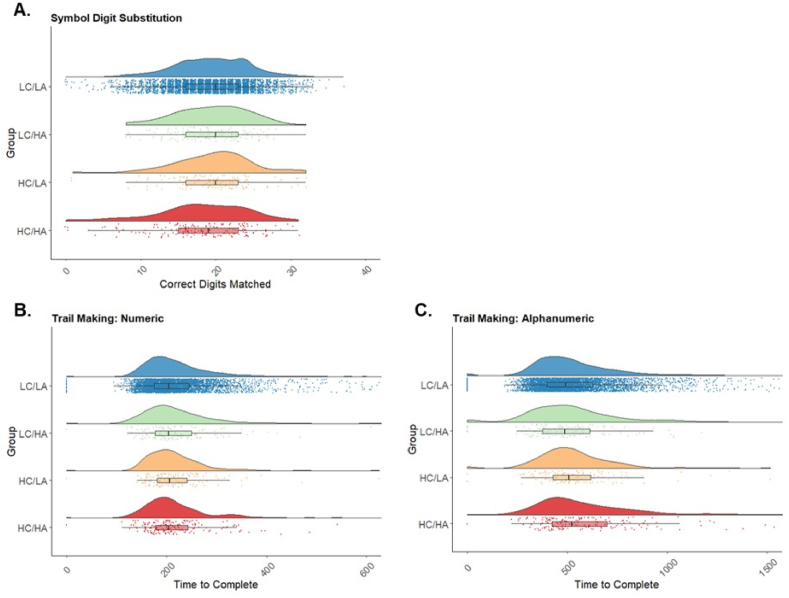
Raincloud plots representing data distributions in each of the four stress groups for (A) correct digits matches for symbol digit substitution task, (B) duration to complete the numeric trail making task (C) duration to complete the alphanumeric trail making task. Box plots represent median and interquartile ranges of these values. Low Childhood and Low Adulthood = LC/LA, Low Childhood and high Adulthood = LC/HA, High Childhood and Low Adulthood = HC/LA and High Childhood and High Adulthood = HC/HA.

ANCOVA tests were used to identify group differences in performance on all three cognitive tasks (FDR corrected; Fig. 5). Age was a significant covariate for all cognitive tasks. Sex was however only a significant covariate for the numeric trail making task.

Numeric trail making performance also revealed a significant effect of group (F(3, 4891) = 3.75, *p* = .01, *η*_*p*_^*2*^ = 0.0023), however *post hoc* analyses show only a non-significant trend for worse performance in the High childhood/High adulthood than the Low childhood/Low adulthood group (*p* = .06, 2.29% increase in mean time to complete).

In contrast, alphanumeric trail making performance also showed group differences (F(3,4891) = 6.47, *p* = .0003, *η*_*p*_^*2*^ = 0.004), but with significantly worse performance in the High childhood/High adulthood than Low childhood/Low adulthood (*p* < .001, 10.28% increase in mean time to complete) and Low childhood/High adulthood groups (*p* = .02, 15.3% increase in mean time to complete).

Symbol digit substitution performance differed by group (F[3, 4891] = 10.86, *p* < .0001, *η*_*p*_^*2*^ = 0.0067). *Post hoc* pairwise comparisons revealed worse performance in High childhood/High adulthood than Low childhood/Low adulthood (*p* < .0001, 6.67% reduction in mean score) and High childhood/Low adulthood stress (*p* = .005, 8.14% reduction in mean score) groups.

Regressions analyses using all stress and cognitive function data from the UK Biobank were performed separately for males and females. Age was found to be a significant predictor of cognitive performance for all tasks with the exception of word production. For both males and females, both childhood and adulthood stress were significant predictors of performance on all tasks except word production (see Table 4, Table 5).

**Table 4:** Sample size and multiple regression outputs for cognitive tasks in relation to Childhood and Adulthood in the male sample.

		Childhood stress		Adulthood stress		Overall model fit	
Task	N	β	p	β	p	R^2^	p
Symbol digit substitution	9671	−0.043	<.001*	−0.081	<.001*	0.210	<.001*
Trail making (numeric)	9750	0.014	.204	0.075	<.001*	0.095	<.001*
Trail making (alphanumeric)	9750	0.032	.003*	0.076	<.001*	0.082	<.001*
Numeric memory	7086	−0.043	.001*	−0.087	<.001*	0.016	<.001*
Word production	353	−0.046	.402	−0.051	.357	−0.001	.457
Prospective memory	23847	0.020	.005*	0.036	<.001*	0.004	<.001*

**Table 5:** Sample size and multiple regression outputs for cognitive tasks in relation to Childhood and Adulthood in the female sample.

		Childhood stress		Adulthood stress		Overall model fit	
Task	N	β	p	β	p	R^2^	p
Symbol digit substitution	10763	−0.048	<.001*	−0.048	<.001*	0.196	<.001*
Trail making (numeric)	10848	0.033	.001*	0.024	.017*	0.078	<.001*
Trail making (alphanumeric)	10848	0.035	.001*	0.041	<.001*	0.085	<.001*
Numeric memory	8839	−0.037	.002*	−0.072	<.001*	0.014	<.001*
Word production	502	−0.024	.618	−0.072	.132	0.001	.301
Prospective memory	30589	0.024	<.001*	0.041	<.001*	0.005	<.001*

### 3.6 Impact of stress on mental health

Multiple regressions were used to assess the relationship between Childhood stress, Adulthood stress and mental health outcomes between males and females separately. In the male sample (*N* = 64453), stress during childhood and adulthood was linked to the number of diagnosed mental health outcomes, with the overall model (F[2, 64450] = 1391.03, p < .001), explaining 4.1% of the variance. When considering the impact of each timing of stress separately, the number of diagnosed mental health outcomes in men was positively associated with childhood stress (β = 0.147, p < .001) and adulthood stress (β = 0.101, p < .001).

In the female sample (*N* = 81755), the overall regression model was significant (F[2, 81752] = 3324.24, p < .001) with stress during Childhood and Adulthood explaining 7.5% of the variance in mental health outcomes. The number of diagnosed mental health conditions in females was positively associated with childhood stress (β = 0.182, p < .001) and adulthood stress (β = 0.150, p < .001).

### 3.7 Mediating effects of brain structure changes

Last, we conducted post hoc analyses to assess whether there was a mediating effect of the brain structure alterations on the relationship between stress (either childhood or adulthood) and the cognitive and mental health outcomes. We used the PROCESS model in R (Hayes, 2012), which uses the product of coefficient approach and bootstrapping to examine the indirect effect. Confidence intervals that do not contain zero are considered significant indirect effects.

For males, we found that OD in the left posterior thalamic radiation and ISOVF in the right cingulum of the hippocampus were significant mediators of the relationship between adulthood stress and performance on the trail making and symbol digit substitution tasks (Table 6). These brain microstructure changes were not mediators of the relationship between adulthood stress and mental health outcomes.

**Table 6:** Outcomes from the mediation analysis using PROCESS to assess the mediation effect of significant brain structure changes related to Adulthood stress (IV) on cognitive ability (DV) in males.

Mediator (Diffusion measure)	Dependent Variable (Cognitive Task)	Direct effect	Indirect effect	95% Confidence Intervals (Upper & Lower Bounds)
**OD in left posterior thalamic radiation**	Symbol digit substitution	−0.205	−0.010	−0.020, −0.002
	Trail making (numeric)	2.746	0.126	0.027, 0.234
	Trail making (alphanumeric)	8.542	0.396	0.070, 0.739
**ISOVF in right cingulum of the hippocampus**	Symbol digit substitution	−0.208	−0.008	−0.015, - 0.002
	Trail making (numeric)	2.788	0.083	0.021, 0.162
	Trail making (alphanumeric)	8.706	0.232	0.052, 0.466

For females, FA, MD and ICVF in the left and right posterior thalamic radiation, and MD and ICVF in the left cingulum of the hippocampus were significant mediators of the relationship between childhood stress and performance on the trail making and symbol digit substitution tests (Table 7). FA and ICVF in the left and right posterior thalamic radiations, and MD in the right posterior thalamic radiation were significant predictors of the relationship between childhood stress and the number of diagnosed mental health outcomes in women.

**Table 7:** Outcomes from the mediation analysis using PROCESS to assess the mediation effect of significant brain structure changes related to Childhood stress (IV) on cognitive ability (DV).

Mediator (Diffusion measure)	Dependent Variable (Cognitive Task or Mental Health Outcomes)	Direct effect	Indirect effect	95% Confidence Intervals (Upper & Lower Bounds)
**FA in left posterior thalamic radiation**	Symbol digit substitution	−0.094	−0.012	−0.021, −0.004
	Trail making (numeric)	0.858	0.127	0.04, 0.214
	Trail making (alphanumeric)	4.115	0.38	0.128, 0.645
	Mental Health Outcomes	0.081	0.0003	0.0001, 0.0006
**FA in right posterior thalamic radiation**	Symbol digit substitution	−0.093	−0.014	−0.022, −0.005
	Trail making (numeric)	0.85	0.136	0.052, 0.224
	Trail making (alphanumeric)	4.008	0.486	0.193, 0.815
	Mental Health Outcomes	0.081	0.0003	0.0001–0.0006
**MD in left cingulum of the hippocampus**	Symbol digit substitution	−0.104	−0.002	−0.0047, −0.0002
	Trail making (numeric)	0.957	0.028	0.0034, 0.06
	Trail making (alphanumeric)	4.428	0.066	0.0006, 0.165
**MD in right posterior thalamic radiation**	Symbol digit substitution	−0.099	−0.007	−0.014, −0.001
	Trail making (numeric)	0.905	0.08	0.01, 0.158
	Trail making (alphanumeric)	4.283	0.212	0.027, 0.414
	Mental Health Outcomes	0.081	0.0002	<0.001–0.0004
**ICVF in left cingulum of the hippocampus**	Symbol digit substitution	−0.102	−0.004	−0.008, −0.0007
	Trail making (numeric)	0.952	0.033	0.003, 0.075
	Trail making (alphanumeric)	4.374	0.12	0.021, 0.262
**ICVF in left posterior thalamic radiation**	Symbol digit substitution	−0.095	−0.011	−0.018, −0.0052
	Trail making (numeric)	0.858	0.127	0.058, 0.202
	Trail making (alphanumeric)	4.168	0.326	0.144, 0.542
	Mental Health Outcomes	0.081	0.0004	0.0001–0.0007
**ICVF in right posterior thalamic radiation**	Symbol digit substitution	−0.096	−0.011	−0.017, 0.005
	Trail making (numeric)	0.87	0.115	0.052, 0.187
	Trail making (alphanumeric)	4.15	0.342	0.155, 0.565
	Mental Health Outcomes	0.081	0.0004	0.0001–0.0007

## 4 Discussion

In this study we examined the association between major stressors at different life stages and the brain's grey matter macrostructure and white matter microstructure. We then tested the links between these stressors and behaviour and life outcomes – particularly cognitive performance and mental health outcomes. We demonstrate that stress during both adulthood and childhood is linked to differences in the microstructure of the bilateral posterior thalamic radiation. This was supported using regression analyses, additionally revealing that microstructural changes in the hippocampal cingulum and posterior thalamic radiations were associated with stress at different stages of the lifespan, with distinct patterns for males and females. More specifically, brain microstructure alterations were linked to childhood stress in females, but to Adulthood stress in males. In both males and females, stress during Childhood and Adulthood was associated with cognitive abilities and mental health outcomes. Intriguingly, the observed microstructural changes were mediators of the relationship between stress and cognitive ability and mental health outcomes, again with distinct patterns between men and women.

A key finding from our results is that the specific associations between stress throughout the lifespan and the brain's microstructure is likely to be sex-dependent. These findings are in line with previous literature, which reports reduced FA and increased MD in limbic regions (and connecting white matter) of females only (Kranz et al., 2017; Sheikh et al., 2014). We also demonstrated an association between females who had experienced more childhood stress and changes in the microstructure of the posterior thalamic radiation and cingulum of the hippocampus, indicating effects on thalamic brain structure and outputs. It has previously been argued that stressful experiences in females have a greater detrimental effect on brain structure than in males (Seeman et al., 2001; Kudielka et al., 2004). This may occur through interactions between stress-related hormones and oestrogen. This is supported by evidence that treatment with oestrogen is associated with decreased FA and increased MD in similar brain regions (Kranz et al., 2017). How oestrogen interacts with cortisol to affect brain microstructure however needs careful study.

Our ANCOVA models suggested that cognitive performance was worse in those with a history of both childhood and adulthood stress. Unlike brain structure, these effects did not differ much between males and females. These findings are in line with previous research regarding both acute and chronic effects of stress on cognition (Boals and Banks, 2012; Wolf, 2003). Stress, particularly during adulthood, was associated with worse performance on alphanumeric trail making tasks. This finding links with previous evidence suggesting that stress during adulthood impairs processing speed, which underpins trail making task performance (Aldwin and Levenson, 2001; MacPherson et al., 2017). Higher stress throughout the lifespan, but particularly within childhood, was associated with worse symbol digit substitution performance. In the wider sample, our regression analyses confirmed that stress during childhood and adulthood was linked to performance alterations on these tasks, highlighting the negative impact of stress at distinct stages of the lifespan on cognitive ability.

Although the impact of stress on cognitive ability did not differ between sexes, the specific brain microstructural changes related to stress (during childhood for females and adulthood for males) were shown to be mediators for the relationship between stress and cognitive performance. Previous research, in both humans and other mammalian species, has suggested that stress at different critical periods across the lifespan may lead to unique and sex specific effects on both brain structure and cognitive ability (Bale and Epperson, 2015). To further this, our finding suggests that changes in cognitive abilities due to stress are, at least in part, underpinned by these stress related alterations in brain structure, however further, more detailed analysis is required to understand these mechanisms in more depth.

As expected, we show clear evidence linking increased levels of both Child- and Adulthood stress with increased numbers of mental health diagnoses. Although this effect is clear in both males and females it is more pronounced, with greater variance explained, in females. In line with this, it has previously been reported that females show higher frequencies of stress-related affective and mental health disorders (Bale and Epperson, 2015; Stroud et al., 2002; Kessler, 2003; Kessler et al., 2015). In the brain, microstructural alterations within the posterior thalamic radiation have previously been associated with major depressive disorder (Liao et al., 2013). The mediation analyses conducted in this study support this possible link by demonstrating a mediating effect of posterior thalamic radiation microstructure on the relationship between childhood stress and the number of diagnosed mental health conditions, specifically in females. Further work can assess at what life stage these changes start to occur.

Our study is, however, not without limitations. Only a subsection of UK Biobank participants had completed neuroimaging (*N* = 5481) and cognitive (*N* = 4897) measures. While we were not testing the same participants in each analysis, the considerable sample sizes should allow for robust conclusions to be drawn. There were fewer participants with high levels of child- and adulthood stress than those with low levels of stress throughout the lifespan. This may reflect the low frequency of these extreme adverse events but may also reflect the potential lack of representation within the biobank of individuals from deprived communities where these stressors may be more prevalent. Similarly, there were comparably greater numbers of females in the high stress groups, reflecting the greater likelihood of experiencing abuse and control in women (Walby and Towers, 2018; Dobash and Dobash, 2004). To account for this, we used sex as a covariate where appropriate and analysed sex-differences in our sample. The UK biobank is however, also a population based sample of largely white European descent. Future work should examine whether the findings hold in other ethnic and cultural groups, to understand the influences these may have.

Additionally, the comparison of macro- and microstructural measures come from different areas (macrostructure in grey matter and microstructure in closely related white matter tracts) making it difficult to compare the two directly. Any differences observed may also reflect differences in location. Future research can directly compare macro- and microstructural changes in the same regions. Another point to consider is that in these analyses we focussed on available brain regions that form the limbic system. Our analyses were to an extent preregistered, as we specifically requested data from these regions from the UK biobank – and had no access to data from other brain regions. Future studies could use whole brain analyses, such as voxel-based morphometry for grey matter volumes or tract-based spatial statistics for white matter metrics, to look for other regions associated with stressful life events. A further limitation concerns our definitions of childhood and adulthood stress. The UK Biobank conducted no specific measure of child and adult life stress. The measure of childhood stress was based on the CTS-5 (Glaesmer et al., 2013), however this may not reflect all possible experiences of stress in childhood. We also used the available data to select comparable questions relating to adult life experiences. Finally, the UK Biobank is a cohort of adults aged 40–69 at entry. It was therefore not possible to examine the effects of stressors at all specific ages across the lifespan, such as any nonlinear effects (e.g. through effects of stress at sensitive developmental periods).

In the present study, we demonstrate that high levels of stress at different points in the lifespan is linked to specific brain changes, and that this may differ between men and women. We also show links between stress during these life stages and both cognitive performance and the number of diagnoses of mental health conditions. These findings shed light on how stressful experiences throughout the lifespan may impact upon the brain, cognition, and mental health outcomes, and how the effects may differ between men and women.

## Disclosures

No conflicts of interest or financial disclosures.

## CRediT authorship contribution statement

**Elizabeth McManus:** Conceptualization, Software, Formal analysis, Writing – original draft. **Hamied Haroon:** Software, Writing – review & editing. **Niall W. Duncan:** Formal analysis, Writing – review & editing. **Rebecca Elliott:** Conceptualization, Writing – review & editing. **Nils Muhlert:** Supervision, Conceptualization, Writing – review & editing.

## Declaration of competing interest

None.

## Figures and Tables

**Table:** 

Field ID	Question/Statement	Reverse Coding Used?	Prefer not to answer	Never True (0)	Rarely True (1)	Sometimes True (2)	Often True (3)	Very Often True (4)
20487	Felt hated by a family member as a child	No	454	132,346	9540	10,432	2502	2074
20488	Physically abused by family as a child	No	358	127,194	16,844	10,614	1424	914
20489	Felt loved as a child	Yes	628	2311	7325	25,660	39,848	81,576
20490	Sexually molested as a child	No	1852	141,849	7186	5002	797	662
20491	Someone to take to doctor when needed as a child	Yes	1074	3378	1305	4322	16,722	130,547

**Table:** 

Field ID	Question/Statement	Reverse Coding Used?	Prefer not to answer	Never True (0)	Rarely True (1)	Sometimes True (2)	Often True (3)	Very Often True (4)
20521	Belittlement by partner or ex-partner as an adult	No	485	119,234	14,964	15,724	3669	3272
20522	Been in a confiding relationship as an adult	Yes	4127	14,051	7401	27,692	26,848	77,229
20523	Physical violence by a partner or ex-partner as an adult	No	491	136,776	9877	7408	1291	1505
20524	Sexual interference by a partner or ex-partner without consent as an adult	No	515	147,762	5028	3035	478	530
20525	Able to pay rent/mortgage as an adult	Yes	2448	5628	1109	4031	11969	132,163

## Data Availability

The authors do not have permission to share data.

## Appendix A

Items used to create child stress scores. Category 145: Online follow up- Mental health- Traumatic events. Field IDs, child stress statement, use of reverse coding and number of each response to the question.

## Appendix B

Two sets of statements and response keys used to create adult stress scores. Category 145: Online follow up- Mental health- Traumatic events. Field IDs, adult stress statement, use of reverse coding and number of each response to the question.
